# Prediction of hot spots in protein–DNA binding interfaces based on discrete wavelet transform and wavelet packet transform

**DOI:** 10.1186/s12859-023-05263-7

**Published:** 2023-04-04

**Authors:** Yu Sun, Hongwei Wu, Zhengrong Xu, Zhenyu Yue, Ke Li

**Affiliations:** 1 School of Information and Computer, Anhui Agricultural University, Hefei, 230036 Anhui China; 2 Information Materials and Intelligent Sensing Laboratory of Anhui Province, Anhui University, Hefei, 230601 Anhui China; 3 Anhui Provincial Engineering Laboratory for Beidou Precision Agriculture Information, Anhui Agricultural University, Hefei, 230036 Anhui China

**Keywords:** Protein–DNA complexes, Hot spot, Synthetic minority over-sampling technique, Discrete wavelet transform, Wavelet packet transform, Light gradient boosting machine

## Abstract

**Background:**

Identification of hot spots in protein–DNA binding interfaces is extremely important for understanding the underlying mechanisms of protein–DNA interactions and drug design. Since experimental methods for identifying hot spots are time-consuming and expensive, and most of the existing computational methods are based on traditional protein–DNA features to predict hot spots, unable to make full use of the effective information in the features.

**Results:**

In this work, a method named WTL-PDH is proposed for hot spots prediction. To deal with the unbalanced dataset, we used the Synthetic Minority Over-sampling Technique to generate minority class samples to achieve the balance of dataset. First, we extracted the solvent accessible surface area features and structural features, and then processed the traditional features using discrete wavelet transform and wavelet packet transform to extract the wavelet energy information and wavelet entropy information, and obtained a total of 175 dimensional features. In order to obtain the best feature subset, we systematically evaluate these features in various feature selection strategies. Finally, light gradient boosting machine (LightGBM) was used to establish the model.

**Conclusions:**

Our method achieved good results on independent test set with AUC, MCC and F1 scores of 0.838, 0.533 and 0.750, respectively. WTL-PDH can achieve generally better performance in predicting hot spots when compared with state-of-the-art methods. The dataset and source code are available at https://github.com/chase2555/WTL-PDH.

**Supplementary Information:**

The online version contains supplementary material available at 10.1186/s12859-023-05263-7.

## Background

Protein–DNA interactions play a vital role in many biological activities, such as DNA replication and repair, gene regulation [1, 2] and transcription. In the protein–DNA interaction interface, a small number of interfacial residues called hot spots contribute more affinity in the interaction [3]. Identification of hot spots plays an important role in exploring the underlying mechanisms and stability of protein–DNA interactions. Alanine scanning mutagenesis [4] has long been used to identify hot spots. At the same time, this experimental method has also been used to explore the mechanism of protein–DNA recognition. Since the high cost, time consuming and labor intensity of the experimental methods, the computational methods provide an alternative way to predict hot spots.

So far, two kinds of computational methods have been used to predict hot spots in protein–DNA complexes. One is based on molecular mechanics, where SAMPDI [5] and PremPDI [6] predict changes in the free energy of protein–DNA binding. SAMPDI-3D [7] is a new version of SAMPDI, which used a gradient lifting decision tree machine learning method to predict protein–DNA binding free energy changes caused by binding proteins and corresponding DNA base mutations. The mCSM-NA [8] approach significantly enhanced the original approach by incorporating pharmacophore modelling and information of nucleic acid properties into graph-based signatures. All the above methods can predict changes of binding free energy in protein–DNA single mutation. mmCSM-NA [9] adapts the well-proven graph-based signature concept based on mCSM-NA and is the first scalable method capable of quantitatively and accurately predicting the effect of multipoint mutations on nucleic acid binding affinity. HISNAPI [10] takes into account the flexibility of protein-nucleic acid complexes by sampling conformations using molecular dynamics simulation, and using empirical force field FoldX to determine the binding energy of wild-type and mutant protein-nucleic complexs. The other is based on machine learning. PrPDH [11] was a method based on 114-dimensional features, which used random forests (VSURF) [12] for feature selection and support vector machine (SVM) [13] as classifier to predict hot spot residues in protein–DNA binding interfaces. inpPDH [14] extracted the traditional features and new interface adjacent property features, used the two-step feature selection strategies for feature selection, and finally built the prediction model based on SVM. sxPDH [15] used supervised isometric feature mapping (S-ISOMAP) [16] and extreme gradient boosting (XGBoost) [17] to predict hot spots in protein–DNA complexes based on features extracted from PrPDH. SPDH [18] was a protein sequence-based hotspot residues prediction method that obtains features from physicochemical property, conservation, solvent accessible surface area, and then feature selection by sequential forward selection (SFS) using SVM as a classifier. PreHots [19] constructed a new dataset consisting of 260 samples from 89 protein–DNA complexes. A total of 157 features were obtained by extracting features such as target residue attributes and network information. Then 19 features were obtained by dimensionality reduction using SFS, and finally an ensemble stacking classifier was employed as the final prediction model. PEMPNI [20] introduced new energy features based on geometric partition and structural features based on interface, and established an integrated model based on energy and non-energy by feature selection and ensemble learning. It can be used to predict changes in the binding free energy of a single mutation. The machine learning-based methods can improve the prediction performance by extracting traditional or new features of protein–DNA complex to predict hot spots. However, some problems still existed, for example, the small scale and the imbalance problem in the data sets have not been solved, and the effective information in the traditional features is not fully utilized.

**Fig. 1:**
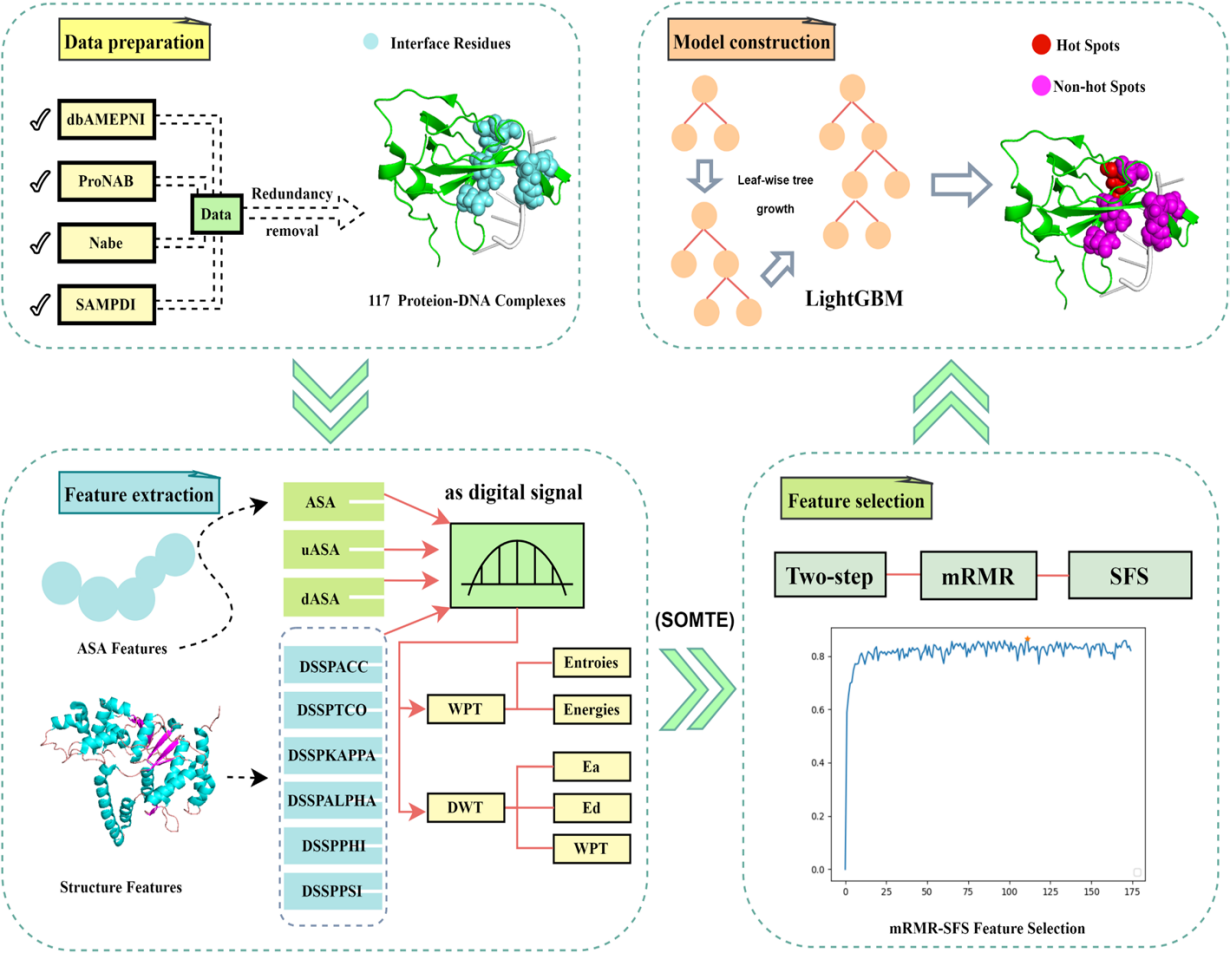
Overall framework of WTL-PDH. First, 117 protein–DNA complexes containing 131 hot spots and 208 non-hot spots in their binding interfaces are collected. The ASA (solvent accessible surface area) and structural feature are extracted. ASA, uASA, dASA and secondary structure feature are treated as four groups of digital signals and DWT and WPT are performed on them to obtain approximate coefficient, detailed coefficients, energy information and wavelet entropy features. The optimal feature subset is obtained using mRMR–SFS. Finally, the final model is constructed based on LightGBM. The predictive performance of our model is evaluated on an independent test dataset

In this work, we proposed a novel method based on discrete wavelet transform (DWT) and wavelet packet transform (WPT) to describe conventional features, termed WTL-PDH, to predict hot spots in protein–DNA binding interfaces. We screened 339 mutations in 117 protein–DNA complexes from dbAMEPNI [21], SAMPDI, Nabe [22], ProNAB [23], and then used Synthetic Minority Over-sampling Technique (SMOTE) [24] to solve the imbalance between positive and negative samples. Firstly, we extracted 43 dimensional traditional features in terms of solvent accessibility surface area, secondary structure, protrusion index and depth index, and hydrogen bond. Then we processed the traditional features by DWT and WPT to obtain 132 dimensional features. We collected a total of 175 dimensional features. To improve the prediction performance, we used a two-step feature selection strategy to obtain 15 best features. Finally, we used light gradient boosting machine(LightGBM) [25] to build the prediction model. To demonstrate its effectiveness, we compared WTL-PDH with the state-of-the-art methods in an independent test set. WTL-PDH achieved generally better performance in predicting hot spots, with an F1 score of 0.766 and an AUC of 0.852 on the training set, as well as an F1 score of 0.750 and an AUC of 0.838 on the test set. The workflow diagram of WTL-PDH is shown in Fig. 1. Both the data and source code are available for download from: https://github.com/chase2555/WTL-PDH.

## Results and discussion

### Comparison of different imbalance data processing algorithms

At present, the data of protein–DNA binding interface residues are less than those of the protein–protein binding interface residues, and the positive and negative samples are unbalanced. More negative samples will lead to a preference for negative samples in the model training process, which is detrimental to model construction. To balance the data, the SMOTE algorithm was used over the training set data to produce a few (positive) class samples, i.e., hot spots. In order to investigate the contribution of the SMOTE method to the prediction performance, three imbalanced data processing algorithms were compared: SMOTE, Balanced by Random Repeat Oversampling (simple replication operation), and adaptive synthetic (ADASYN) [26].ADASYN is an adaptive synthetic sampling method similar to SMOTE, but based on a local distribution estimate of the oversampled class, then generates a different number of samples. As shown in Table 1, there is a significant improvement in the performance of the model trained using the balanced data compared to the model trained using the initial unbalanced data. The model performance SPE = 0.959 and SEN = 0.175 when based on unbalanced data set, indicating that the predicted values always lean toward negative samples due to the large number of negative samples.. Its AUC is only 0.734, and generalization ability of the model is poor. After the SMOTE operation, the model performance AUC is 0.852 and F1 is 0.766, and SMOTE achieved better results under comprehensive evaluation. We believe the reason for the improvement is that the data imbalance makes the model construction mainly dominated by negative samples, which is not conducive to model training. The balanced data obtained by SMOTE processing are more favorable for model construction.

**Table 1:** Effect of different data processing methods on the training set for the model

Data	SEN	SPE	PRE	F1	MCC	ACC	AUC
Balanced by SMOTE	**0.794**	0.735	**0.749**	**0.766**	**0.537**	**0.765**	**0.852**
Balanced by random repeat oversampling	0.759	0.665	0.701	0.724	0.431	0.712	0.780
Imbalanced	0.175	**0.959**	0.365	0.236	0.170	0.667	0.734
ADASYN	0.727	0.576	0.659	0.681	0.314	0.654	0.723

The highest value in each column is shown in bold

### Evaluation of different feature selection methods

We compared six feature selection methods based on LightGBM classification model, including mRMR, SFS, RF, SVM–RFE, mRMR–SFS, and RF–SFS. Table 2 shows the performance of models on different feature selection methods. It can be seen that PRE based on RF–SFS model is almost the same as that based on mRMR–SFS model, but other indicators based on mRMR–SFS model are significantly higher than those of RF–SFS model. The model using mRMR–SFS approach yielded the best performance with an AUC of 0.852. In contrast, the AUC scores generated by the other five methods are relatively low. mRMR–SFS method can find and rank a set of features from the original feature set that are most relevant to the sample label but least relevant to each other. Then, SFS selects one feature at a time to add to the feature subset, which can make the model achieve the best performance. Therefore, mRMR-SFS method was selected as the optimal feature selection method of the prediction model.

**Table 2:** Performance comparison of different feature selection methods on the training set

Method	SEN	SPE	PRE	F1	MCC	ACC	AUC
mRMR-SFS (15)	**0.794**	**0.735**	0.749	**0.766**	**0.537**	**0.765**	**0.852**
mRMR (9)	0.706	0.724	0.740	0.709	0.444	0.715	0.792
SFS (13)	0.688	0.706	0.713	0.691	0.405	0.697	0.787
RF (18)	0.759	0.712	0.729	0.738	0.479	0.735	0.831
RF–SFS (14)	0.724	0.735	**0.750**	0.722	0.476	0.729	0.829
SVM–RFE (20)	0.600	0.582	0.595	0.592	0.186	0.591	0.591

The highest value in each column is shown in bold. The numbers in parentheses represent the dimensionality of the features after dimensionality reduction

### The importance of different features

Through mRMR–SFS, we selected 15 optimal features (Table S1 in Additional file 1), among which two features belong to ASA, one to secondary structure feature and two to DPX, CX features. The other 10 Wavelet features were newly extracted by DWT and WPT, which were ASA_Wavelet(7 dimensions) extracted from ASA and DSSP_Wavelet(3 dimensions) extracted from secondary structure, respectively. In order to better understand the contribution of different categories of features to the prediction performance, we removed the features of different categories in turn and compared their cross-validation performance, as shown in Table 3. When Wave features were removed, all aspects of WTL-PDH’s evaluation indicators decreased significantly, and prediction performance of the model decreased, which emphasized the importance of our newly extracted Wave features. In addition, ASA_Wavelet accounted for a large proportion after feature selection, and after it was removed, the MCC and AUC of the model decreased by 34.6% and 16.8%. It can be seen that these seven features show more contribution correctly predicting hot spot residues. When only ASA_Wavelet is included, the AUC reaches 0.756, and when ASA features are added, the AUC reaches 0.794. ASA original features contained important information to predict hot spot residues. DWT and WPT can analyze the details and various entropy information in digital signals, we fully excavated the important information through DWT and WPT. These results show that Wave features have great contribution to identifying hot spots and non-hot spots, and are complementary to other categories of features, which are helpful to predict the hot spots of protein–DNA complexes.

**Table 3:** Comparison of prediction performance using different feature models on the training set

Features	SEN	SPE	PRE	F1	MCC	ACC	AUC
All features (15)	**0.794**	0.735	0.749	**0.766**	**0.537**	**0.765**	**0.852**
Without-DSSP (13)	0.735	0.741	0.740	0.724	0.491	0.738	0.835
Without-ASA (13)	0.729	0.741	0.742	0.729	0.477	0.735	0.846
ASA_Wavelet (7)	0.700	0.700	0.706	0.696	0.409	0.700	0.756
Without-ASA_Wavelet (8)	0.688	0.653	0.668	0.669	0.351	0.671	0.714
ASA and ASA_Wavelet (9)	0.688	0.706	0.780	0.691	0.401	0.697	0.794
Without-DSSP_Wavelet (12)	0.765	**0.747**	**0.753**	0.754	0.519	0.756	0.828
Without Wavelet features (5)	0.635	0.647	0.641	0.632	0.286	0.641	0.719
Without-DPX and CX (13)	0.718	0.718	0.721	0.711	0.443	0.718	0.793

The highest value in each column is shown in bold. The numbers in parentheses represent the dimensionality of the features after dimensionality reduction

### Performance comparison among different machine learning methods

In order to obtain the most suitable prediction model for hot residues in protein–DNA binding interface, we comprehensively evaluated the model performance of LightGBM, K-nearest neighbor (KNN), logistic regression (LR), SVM, RF and the classic deep learning model CNN(Convolutional Neural Network). To ensure the comparability of the results, the parameters of each machine learning method were adjusted. Additional file 1: Table S2 shows the performance comparison of the five machine learning classifiers with tenfold cross-validated on the training set. LightGBM is superior to the other four machine learning methods on the training set (SEN = 0.794, SPE = 0.735, F1 = 0.766, MCC = 0.537, ACC = 0.765, AUC = 0.852). Although RF has a slight advantage in PRE, LightGBM model is more suitable for constructing protein–DNA hotspot residues prediction model.

### Comparison with other methods

To accurately evaluate WTL-PDH performance, we performed tenfold cross-validation 50 times on the training dataset, and the results are shown in Additional file 1: Table S3. WTL-PDH produced fairly good performance, and the average values of F1, MCC, ACC, AUC were 0. 771, 0.539, 0.768, and 0.851 respectively. These results show that the performance of our model is relatively efficient and reliable.

**Fig. 2:**
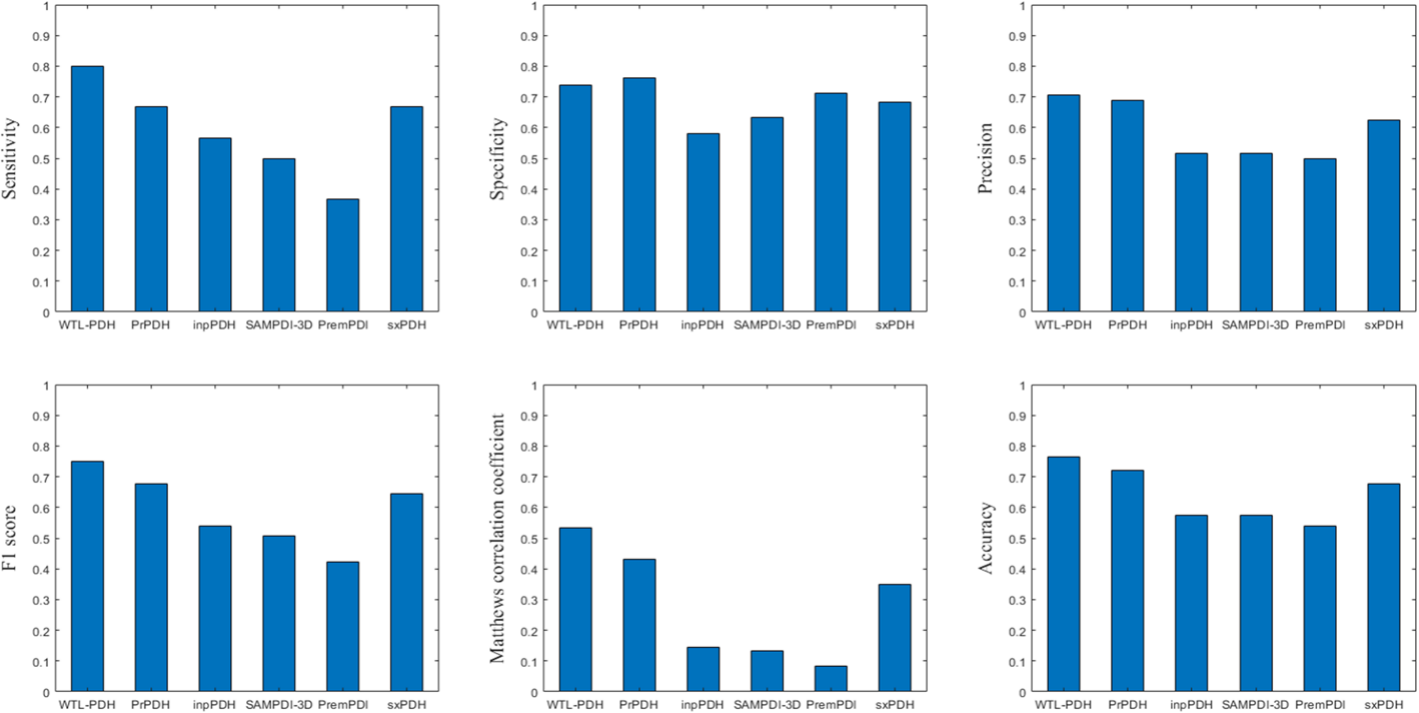
Performance comparisons of WTL-PDH with four other methods on the test set

**Fig. 3:**
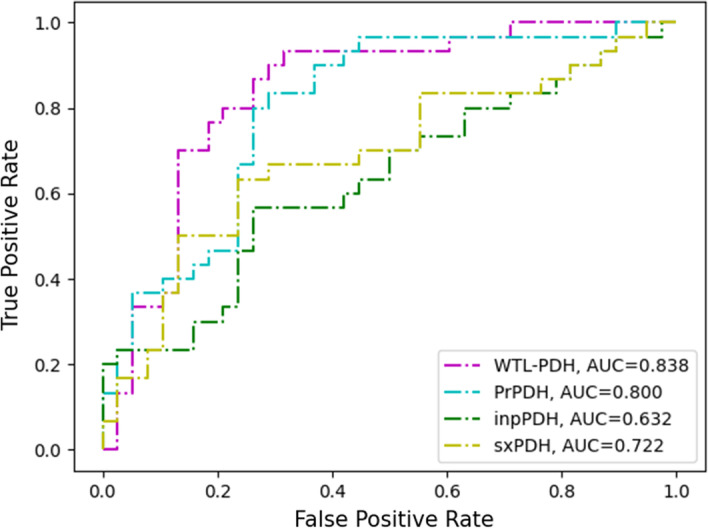
ROC curves of different methods on independent test sets

To further verify the performance of our model, it was compared to state-of-the-art methods including SAMPDI-3D, PremPDI, mmCSM-NA and sxPDH, PrPDH, inpPDH. sxPDH, PrPDH and inpPDH used classification models to distinguish hot spot residues in protein–DNA binding interface, while SAMPDI-3D, PremPDI and mmCSM-NA used regression models to predict changes in protein–DNA binding free energy. Figure 2 shows the performance of WTL-PDH compared to the five methods on the test set. In our method WTL-PDH has SEN = 0.800, PRE = 0.706, F1 = 0.750, MCC = 0.533, ACC = 0.765. Only our SPE is smaller than that of mmCSM-NA, which is due to mmCSM-NA's “preference” for non-hot spots. Since SAMPDI-3D, PremPDI, and mmCSM-NA were predicted by an online server, we plotted ROC curves on independent test sets for the other methods. As shown in Fig. 3, it can be seen that WTL-PDH achieved the best prediction performance with AUC = 0.838. These results indicate that WTL-PDH has impressive performance in predicting hot spots in protein–DNA binding interfaces. The detailed results of the performance comparison are listed in Additional file 1: Table S4.

### Case study

**Fig. 4:**
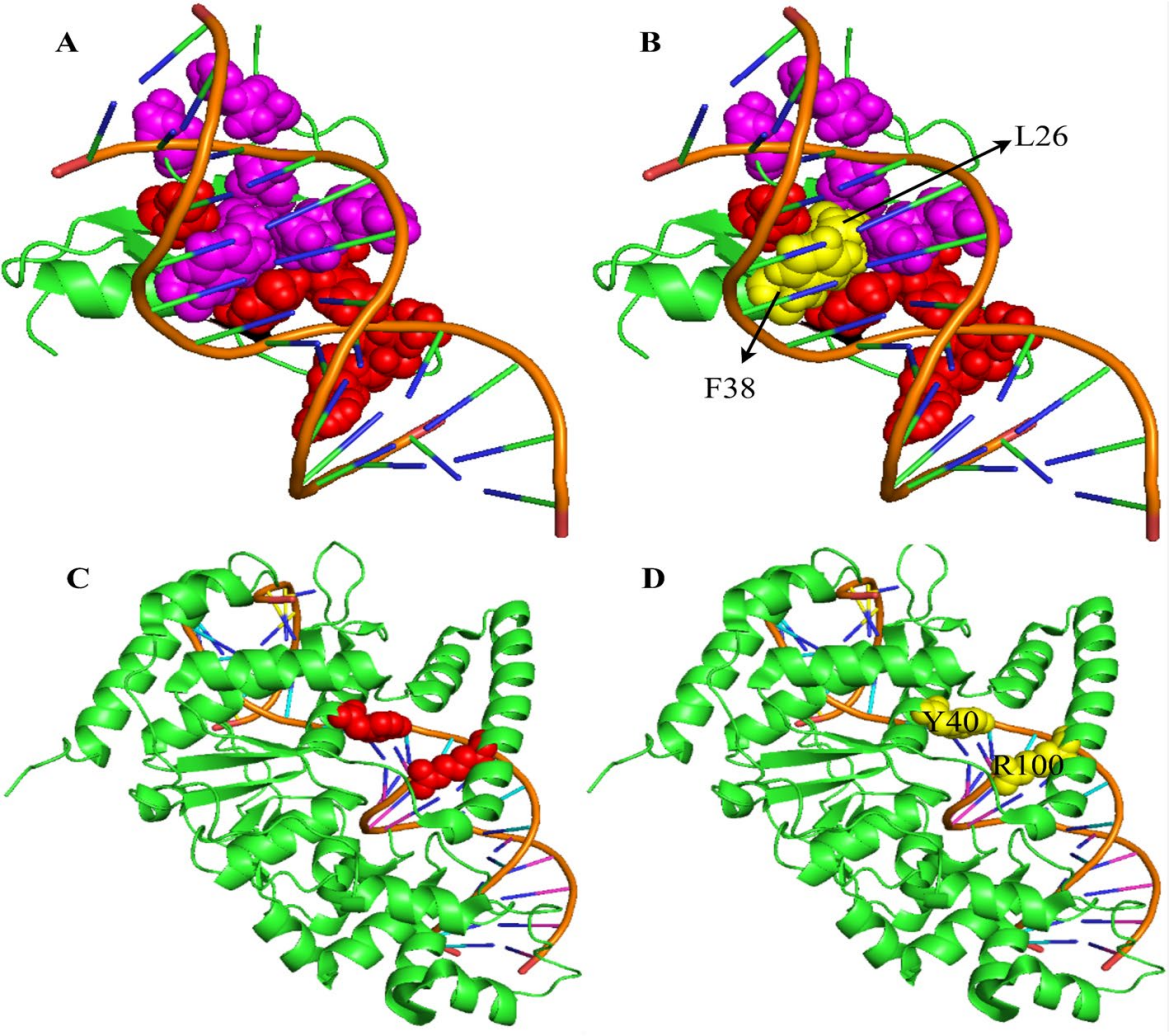
Visualization of hot spots and non-hot spots in 1TN9 using WTL-PDH (**A**) and PrPDH (**B**). Visualization of hot spots and non-hot spots in 3Q8L using WTL-PDH (**C**) and PrPDH (**D**). The following color scheme was used: orange for DNA sequences and green for protein sequences. Red represents correctly predicted hot spots and purple represents correctly predicted non-hot spots. Yellow represents incorrectly predicted hot spots and non-hot spots

As a case study, Fig. 4 shows the predicted results of WTL-PDH for two protein–DNA complexes. The TN916 integrase protein (PDB ID: 1TN9, chain A) binds to DNA through its N-terminal domain [27]. For this complex, a total of five hot spots and eight non-hot spots were found on the protein chain, among which R24, K28, K40, K54 and R55 are hot spots, and R5, T15, S18, R20, K21, L26, F38 and R55 are considered as non-hot spots. The yellow residues represent the residues that were incorrectly predicted. The prediction results for WTL-PDH and PrPDH can be found in Fig. 4A, B. WTL-PDH identifies all hot spots and non-hot spots. PrPDH identified two non-hot spots (L26 and F38) incorrectly. The second one is the crystal structure of human flap nuclease FEN1 (WT) complexed with substrate 5'-flap DNA, SM3 + and K + (PDB ID: 3Q8L, chain A) [28]. There are two hot spots (Y40 and R100) in the protein chain. As shown in Fig. 4C, D, WTL-PDH correctly identified both hot spots, while PrPDH made all predictions incorrectly.

## Conclusions

In this work, we propose a novel method named WTL-PDH to distinguish protein–DNA binding hot spots. Based on our previous work, we integrated the recently proposed Nabe and ProNAB to expand our data set, and generated minority (positive) class samples using the SMOTE algorithm to achieve data category balance. In addition to extracting traditional features, we also used DWT and WPT to extract wavelet energy features and wavelet entropy features, and 175-dimensional features were collected. In order to improve the prediction performance of the model, 15 optimal feature subsets were obtained based on the two-step feature selection method of mRMR-SFS. Finally, we built the final prediction model using LightGBM. The results show that the wavelet feature can effectively describe the difference between hot spots and non-hot spots, and can effectively improve the prediction performance of the model. In addition, we compared our model with the existing methods on an independent test set,. The experimental results show that our model is superior to the existing methods in identifying hot spots in protein–DNA binding interfaces. We believe our approach provides new ideas for accurately identifying hot spots.

In our future work, on the one hand, we will try to digitally encode protein sequences and combine them with common digital signal processing methods to develop more efficient and simple prediction methods. On the other hand, we will continue to explore the valid information in the traditional features to make our models more powerful.

## Materials and methods

### Data sets

In this study, compared with our previous work sxPDH [15], two new dataset sources were added, one from Nabe [22] and the other from ProNAB [23]. We collected 1627 mutations in 293 complexes. To eliminate redundancy, proteins with > 40% sequence similarity were deleted by CD-HIT [29]. Interfacial residues with solvent accessible surface area greater than 1 Å were selected using NACCESS [30].

Referring to the previous criteria [11], the interfacial residues with ∆∆G ≥ 1.0 kcal/mol were defined as hot spots and those with less than 1.0 kcal/mol were defined as non-hot spots. Finally, we obtained 117 protein–DNA complexes containing 131 hot spots and 208 non-hot spots. Ninety-two complexes were randomly selected to constitute the training set, including 101 hot spots and 170 non-hot spots. The remaining 25 complexes constitute the test set, which contains 30 hot spots and 38 non-hot spots. The final benchmark data sets used in our study are shown in Table 4.

**Table 4:** The final benchmark data sets used in our study

Data set	Number of mutations	Number of PDBs	Number of hot spots	Number of non-hot spots	Ratio^a^
Training	271	92	101	170	0.594
Test	68	25	30	38	0.789

^a^indicates the ratio of positives to negatives in the training/test set

### Feature extraction

In order to better distinguish hot spots from non-hot spots, we extracted 43-dimensional traditional features, which are solvent accessible surface area features and structural features, respectively. Then, the different classes of features were regarded as a group of digital signals, and 132-dimensional new features were extracted by DWT and WPT. In total, 175 features, whose details are given below.

### Solvent accessible surface area features

Several studies have shown that ASA plays an important role in identifying hot spots in protein–protein and protein–DNA binding interfaces [11, 31, 32]. We used the NACCESS [30] to calculate the absolute ASA and relative ASA (RSA) features under four atomic properties of residues, including all atoms, nonpolar side chains, polar side chains, and all side chains, and obtained a total of 8-dimensional ASA features. The ASA and RSA of these four properties in monomer and complex state were calculated. The relative change in ASA and RSA between the two states were considered as features. A total of 24-dimensional ASA characteristics were quantified.

### Secondary structure features

Definition of Secondary Structure of Proteins (DSSP) [33] was used to calculate the secondary structure features of proteins, which include the carbonyl angles, bond angles, torsion angles, and the number of water molecules. A total of 6-dimensional features were quantified.

### Depth index and protrusion index

Depth index (DPX) and protrusion index (CX) can improve the prediction performance of the model for hot residues [32]. We used PSAIA [34] to calculate two atomic property values of a residue between the bound and unbound states, including the mean value of all atoms and the standard deviation of side chain atoms, a total of 8-dimensional features. The relative changes of DPX and CX between the two states were also calculated respectively, a total of 4-dimensions. In total, 12-dimensional features were quantified.

### Hydrogen bond

Hydrogen bonds affect protein–DNA recognition [14, 19, 35]. Here, we used HBPLUS [36] to calculate the hydrogen bonds of protein–DNA complexes.

### Discrete wavelet transform (DWT)

DWT and WPT have long been used in signal analysis and processing [37, 38], they were time domain analysis methods which can effectively process various types of non-stationary random signals. DWT and WPT have been widely used in image processing and bioinformatics [39–43]. DWT can decompose the original signal into a crude approximation coefficient (lower frequency) and a specific detailed coefficient (higher frequency), and then the approximate coefficients are further decomposed into high and low frequencies [44]. The total number of low and high frequencies is $\mathop 2\nolimits^{n}$ [45] after a signal is decomposed by DWT for n levels, DWT can be expressed by the following equation:

1 $$ Dwt\left( {x,y} \right) = \frac{1}{\sqrt x }\int_{0}^{t} {f\left( t \right)\psi \left( {\frac{t - y}{x}} \right)\mathop d\nolimits_{t} } $$

$x$, $y$ denote the scale and translation variables, respectively.$f\left( t \right)$ is the signal, and $\psi \left( {\frac{t - y}{x}} \right)$ represents the wavelet function at a particular scale $x$ and translation $y$.

In fact, the DWT can be implemented with a low-pass filter $g\left[ k \right]$ and a high-pass filter $h\left[ k \right]$ [46]. The approximation coefficients are obtained by convolving the input signal $f\left( t \right)$ with the scaling filter and performing a dyadic decimation. The approximation coefficients are obtained by convolving the input signal a with the scaling filter and then performing a dyadic decimation. Similarly, the signal $f\left( t \right)$ is convolved with the wavelet filter and then performing a dyadic decimation to produce the detail coefficients. In this way, the signal is decomposed into low-frequency and high-frequency components, as follows:

2 $$ \left\{ \begin{gathered} \mathop E\nolimits_{j,L} \left[ n \right] = \sum\limits_{k} {s\left[ k \right]\; g\left[ {2n - k} \right]} \hfill \\ \mathop E\nolimits_{j,H} \left[ n \right] = \sum\limits_{k} {s\left[ k \right]\; h\left[ {2n - k} \right]} \hfill \\ \end{gathered} \right. $$

where $\mathop E\nolimits_{j,L}$ is the approximate coefficient of the signal, which represents the low frequency component. $\mathop E\nolimits_{j,H}$ is the detailed coefficient, which means the high frequency component, and $s\left[ k \right]$, $j$ are the level of discrete signal and the split scale,. We use Ea, Ed to further characterize the information contained in $\mathop E\nolimits_{j,L}$ and $\mathop E\nolimits_{j,H}$. Ea and Ed are the percentage of energy in the approximate and detailed coefficients, respectively.

Entropy is a measure of the uncertainty of a random variable. It is proposed to solve the problem of quantifying information [47]. Wavelet entropy is usually used to analyze non-stationary signals and can better characterize the information contained in the signal [48]. In order to better describe the information inside the traditional features, we extract five kinds of wavelet entropies from the signal. In the following formula, $s$ is the signal and $\mathop s\nolimits_{i}$ is the coefficient of $s$ in the orthogonal basis.The entropy E must be an additive cost function such that $E\left[ 0 \right] = 0$ and $E\left( s \right) = \sum\nolimits_{i} {E\left( {\mathop s\nolimits_{i} } \right)}$. The five wavelet entropies are described as follows:

The Shannon entropy:

3 $$ \mathop E\nolimits_{s} \left( s \right) = - \sum\nolimits_{i} {\mathop s\nolimits_{i}^{2} \log \left( {\mathop s\nolimits_{i}^{2} } \right)} $$

The logarithm of the “energy” entropy:

4 $$ \mathop E\nolimits_{l} \left( s \right) = \sum\nolimits_{i} {\log \left( {\mathop s\nolimits_{i}^{2} } \right)} $$

The other three wavelet entropies are: compute the threshold entropy of $s$ using a threshold value of 0.2, compute the Sure entropy of $s$ with the threshold equal to 3, compute the norm entropy of $s$ with power equal to 1.3.

ASA has been shown to play an important role in predicting protein–DNA hotspot residues [11, 31, 32]. Therefore we divided the extracted 24-dimensional ASA features into three groups, each containing 8-dimensional features, namely ASA (four attributes of ASA and RSA in the monomeric state), uASA (complex), and dASA (relative change between monomeric and complex states). Similarly, we divided the secondary structure features (6 dimensions) into a single group. These 4 groups of features were treated as 4 channels of digital signals, which were processed by DWT. The wavelet function we selected is db1, which performed three levels of decomposition by DWT. Their Ea (3 dimensions), standard deviation of Ea(1 dimensions), mean of Ea(1 dimensions), Ed(1dimensions) and the above wavelet entropy features(5 dimensions) were calculated respectively. A total of 4 × 11 = 44 dimensional features were obtained based on DWT.

### Wavelet packet transform (WPT)

To obtain more richer information from conventional features, WPT is used to further decompose the detailed information in the high frequency region of the digital signal [38]. The function $\psi \left( n \right)$ is called a wavelet packet with respect to the scale function $\varphi \left( x \right)$. If WPT is used to decompose the signal at the third level [44], the equation can be unified as:

5 $$ \left\{ \begin{gathered} \mathop s\nolimits_{2n} \left( t \right) = \sqrt 2 \sum\limits_{k} {\mathop s\nolimits_{n} \left( {2t - k} \right)h\left( k \right)} \hfill \\ \mathop s\nolimits_{2n} \left( t \right) = \sqrt 2 \sum\limits_{k} {\mathop s\nolimits_{n} \left( {2t - k} \right)g\left( k \right)} \hfill \\ \end{gathered} \right. $$

**Fig. 5:**
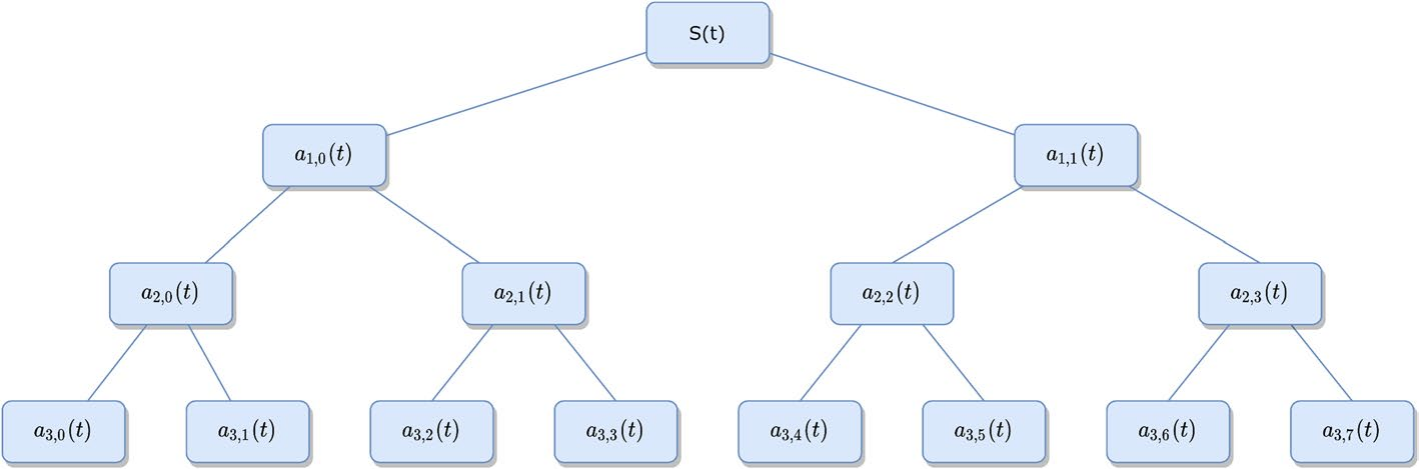
Schematic diagram of WPT with signal undergoing third level splitting

If $\varphi \left( t \right)$ is the wavelet function and $\psi \left( t \right)$ is its corresponding scaling function, when $\mathop s\nolimits_{0} \left( t \right) = \varphi \left( t \right)$ and $\mathop s\nolimits_{1} \left( t \right) = \psi \left( t \right)$, the signal is decomposed as:

6 $$ \left\{ \begin{gathered} \mathop a\nolimits_{i + 1,2n} = \sum\limits_{j} {\mathop a\nolimits_{i,n} \,h\left( {j - 2k} \right)} \hfill \\ \mathop a\nolimits_{i + 1,2n + 1} = \sum\limits_{j} {\mathop a\nolimits_{i,n\,} g\left( {j - 2k} \right)} \hfill \\ \end{gathered} \right. $$

where $\mathop a\nolimits_{i,n}$ represents the wavelet coefficients of level $i$ under $n$ sub-bands,$j$ is the number of wavelet coefficients. As shown in Fig. 5, a WPT that performs the third level of splitting produces a total of 8 sub-bands, each of which covers 1/8 of the frequency information.

Similar to DWT, four groups of features, ASA, uASA, dASA and secondary structure, were treated as digital signals and processed by WPT, and the with wavelet function was db1. We extracted the relative energy (8 dimensions), absolute energy (8 dimensions), absolute energy sum (1 dimension), and wavelet entropy features (5 dimensions) of the terminal nodes in the third layer of the wavelet packet tree. So far, a total of 22 × 4 = 88 dimensions were extracted based on WPT.

### SMOTE algorithm

SMOTE is a modified scheme based on the random oversampling algorithm. The basic idea is to analyze the minority class samples, and then artificially synthesize new samples to add to the data set based on the minority class samples. For each sample X in the minority class, calculate its distance to all samples in the minority class sample set S using Euclidean distance to get its K nearest neighbor. For each randomly selected nearest neighbor $\mathop X\nolimits_{n}$, a new sample is constructed according to the following formula:

7 $$ \mathop X\nolimits_{new} = X + \left| {X - \mathop X\nolimits_{n} } \right| \times rand\left( {0,1} \right) $$

To make the experiment reproducible, put random_rate at 114 in all of the model based on SMOTE.

### Feature selection

Too high feature dimensionality can lead to overfitting of the classifier. For our dataset, 175-dimensional candidate features appear redundant and large. Therefore, feature selection is essential to improve the prediction performance of the classifier. In fact, we adopted a two-step feature selection strategy to remove irrelevant and redundant features. In the first step, we ranked all features using the maximum relevance minimum redundancy (mRMR) [49]. In the second step, we used SFS method to process the mRMR-derived feature sequences to obtain an optimal feature subset. We also compared the results with five common feature selection methods. These methods are random forest (RF) [50], SVM-based recursive feature elimination (SVM–RFE) [51], SFS, mRMR, and RF based on sequential forward selection (RF–SFS) [52].

### Model building

LightGBM has achieved the better results in many Machine Learning challenges. It is a distributed gradient boosting framework based on the decision tree algorithm. To meet the industry's demand for shorter model times, LightGBM uses a histogram-based decision tree algorithm. To avoid overfitting as much as possible, LightGBM includes a parameter that limits the depth of the tree. Compared with XGBoost, it has faster training speed and higher accuracy. On the training set, we use grid search method to adjust its parameters, and obtain the optimal parameters of max_depth = 15, num_leaves = 50, n_estimators = 1000.

### Evaluation criteria

We employed tenfold cross-validation method on the training set for feature selection to obtain the best features and tune the parameters of LightGBM. To evaluate the performance of the model, we used some common evaluation metrics: including sensitivity (SEN), specificity (SPE), precision (PRE), F1 score (F1), accuracy (ACC), and Matthews correlation coefficient (MCC). These measurements are defined as follows:

8 $$ SEN = \frac{TP}{{TP + FN}} $$

9 $$ SPE = \frac{TN}{{TN + FP}} $$

10 $$ PRE = \frac{TP}{{TP + FP}} $$

11 $$ \mathop F\nolimits_{1} = \frac{2 \times SEN \times PRE}{{SEN + PRE}} $$

12 $$ ACC = \frac{TP + TN}{{TP + TN + FP + FN}} $$

13 $$ MCC = \frac{TP \times TN - FP \times FN}{{\sqrt {\left( {TP + FP} \right)\left( {TP + FN} \right)\left( {TN + FP} \right)\left( {TN + FN} \right)} }} $$

where TP, FP, TN and FN represent the number of true positive (correctly predicted hot spot residue), false positive (non-hot spot residue incorrectly predicted as hot spot), true negative (correctly predicted non-hot spot residue) and false negative (hot spot residue incorrectly predicted as non-hot spot), respectively. For completeness, we also calculated the area of the ROC curve called AUC to evaluate our performance.

## Supplementary Information

**Additional file 1**.** Table S1**: The rankings of the 19 selected features.** Table S2**: Comparison of the performance of different machine learning classifiers on the training set.** Table S3**: WTL-PDH on training dataset 50 times 10-fold cross-validation results.** Table S4**: Performance comparison of different methods on the test set.

## Data Availability

The data and python code of sxPDH are freely available via GitHub: https://github.com/chase2555/WTL-PDH.

## Abbreviations

SMOTE: Synthetic minority over-sampling technique
DWT: Discrete wavelet transform
WPT: Wavelet packet transform
LightGBM: Light gradient boosting machine
mRMR: Maximum relevance minimum redundancy
SFS: Sequential forward selection
RF–SFS: RF based on sequential forward selection
SVM–RFE: SVM-based recursive feature elimination
SEN: Sensitivity
SPE: Specificity
PRE: Precision
F1: F1 score
ACC: Accuracy
MCC: Matthews correlation coefficient
AUC: The area under the ROC curve
