# Supplementary material for: Prediction of hot spots in protein–DNA binding interfaces based on discrete wavelet transform and wavelet packet transform
Source: BMC Bioinformatics. 2023 Apr 4;24:129. doi: 10.1186/s12859-023-05263-7 (PMC10074722; doi:10.1186/s12859-023-05263-7)
Supplement: Supplementary file 1 — Additional file 1. Table S1: The rankings of the 19 selected features. Table S2: Comparison of the performance of different machine learning classifiers on the training set. Table S3: WTL-PDH on training dataset 50 times 10-fold cross-validation results. Table S4: Performance comparison of different methods on the test set. [file 12859_2023_5263_MOESM1_ESM.docx]

**Table S1.** The rankings of the 19 selected features

| **Number** | **Symbol** | **Feature description** |
| --- | --- | --- |
| 1 | **u-Non-polar-ABS** | Nonpolar side chains of the absolute ASA in the unbound state |
| 2 | **d-Non-polar-ABS** | Changes in the non-polar side chains of the absolute ASA in the bound and unbound states |
| 3 | **ASA_node2_relative_energy** | Relative energy of the second node in the third layer after WPT processing of ASA |
| 4 | **uASA_node5_REF _energy** | Relative energy of the 5th node after WPT processing by uASA |
| 5 | **uASA_node8_ABS_energy** | Absolute energy of the 8th node after WPT processing by uASA |
| 6 | **uASA_sum_absolute_energy** | Total absolute wavelet energy of the third layer of uASA after WPT processing |
| 7 | **dASA_node8_relative_erergy** | Relative energy of the 8th node after WPT processing by uASA |
| 8 | **uASA_Ea_3** | The third energy percentage of the approximation coefficients of the wavelet of the third layer after DWT by uASA |
| 9 | **dASA_Ea_2** | The second energy percentage of the approximate coefficients of the wavelet of the third layer after DWT of the dASA |
| 10 | **DSSPPSI** | IUPAC peptide backbone torsion angles PSI |
| 11 | **dssp_b_shannon** | Shannon entropy of secondary structural features after WPT |
| 12 | **dssp_b_log_energy** | Log erergy entropy of secondary structural features after WPT |
| 13 | **dssp_sure** | Shannon entropy of secondary structural features after DWT |
| 14 | **d-average-DPX** | Depth index Change in average value of all atoms in bound and unbound states |
| 15 | **d-s-ch-avg-CX** | Protrusion index change in standard deviation of all side chain atoms in bound and unbound states |

**Table S2.**Comparison of the performance of different machine learning classifiers on the training set

| Classifier | SEN | SPE | PRE | F1 | MCC | ACC | AUC |
| --- | --- | --- | --- | --- | --- | --- | --- |
| LightGBM | **0.794** | **0.735** | 0.749 | **0.766** | **0.537** | **0.765** | **0.852** |
| KNN | 0.588 | 0.718 | 0.687 | 0.624 | 0.317 | 0.653 | 0.653 |
| LR | 0.665 | 0.641 | 0.661 | 0.658 | 0.311 | 0.653 | 0.653 |
| SVM | 0.674 | 0.512 | 0.593 | 0.636 | 0.212 | 0.603 | 0.603 |
| RF | 0.758 | 0.759 | **0.763** | 0.747 | 0.517 | 0.753 | 0.753 |
| CNN | 0.653 | 0.529 | 0.666 | 0.559 | 0.230 | 0.591 | 0.673 |

The highest value in each column is shown in bold.

**Table S3.** WTL-PDH on training dataset 50 times 10-fold cross-validation results

| Method | SEN | SPE | PRE | F1 | MCC | ACC | AUC |
| --- | --- | --- | --- | --- | --- | --- | --- |
| WTL-PDH | 0.782 | 0.753 | 0.766 | 0.771 | 0.539 | 0.768 | 0.851 |

**Table S4.** Performance comparison of different methods on the test set

| Method | SEN | SPE | PRE | F1 | MCC | ACC | AUC |
| --- | --- | --- | --- | --- | --- | --- | --- |
| WTL-PDH | **0.8** | 0.737 | **0.706** | **0.750** | **0.533** | **0.765** | **0.838** |
| sxPDH | 0.733 | 0.711 | 0.667 | 0.698 | 0.441 | 0.721 | 0.722 |
| PrPDH | 0.667 | 0.763 | 0.690 | 0.678 | 0.432 | 0.721 | 0.800 |
| inpPDH | 0.567 | 0.579 | 0.515 | 0.54 | 0.145 | 0.574 | 0.632 |
| SAMPDI-3D | 0.5 | 0.632 | 0.517 | 0.508 | 0.132 | 0.574 | 0.574 |
| PremPDI | 0.367 | 0.711 | 0.5 | 0.423 | 0.082 | 0.539 | 0.423 |
| mmCSM-NA | 0 | **0.974** | 0 | 0 | -0.109 | 0.544 | 0.487 |

The highest value in each column is shown in bold.
